# “Being the main character but not always involved in one’s own care transition” - a qualitative descriptive study of older adults’ experiences of being discharged from in-patient care to home

**DOI:** 10.1186/s12913-024-11039-3

**Published:** 2024-05-02

**Authors:** Emelie Ingvarsson, Kristina Schildmeijer, Heidi Hagerman, Catharina Lindberg

**Affiliations:** Department of Health and Caring Sciences, Linnaeus University, Universitetsplatsen 1 392 31, Kalmar, Växjö, Sweden

**Keywords:** Care transition, Chronic disease, Coordinated care, Discharge, Experiences, Interviews, Older adults

## Abstract

**Background:**

The growing number of older adults with chronic diseases challenges already strained healthcare systems. Fragmented systems make transitions between healthcare settings demanding, posing risks during transitions from in-patient care to home. Despite efforts to make healthcare person-centered during care transitions, previous research indicates that these ambitions are not yet achieved. Therefore, there is a need to examine whether recent initiatives have positively influenced older adults’ experiences of transitions from in-patient care to home. This study aimed to describe older adults’ experiences of being discharged from in-patient care to home.

**Methods:**

This study had a qualitative descriptive design. Individual interviews were conducted in January–June 2022 with 17 older Swedish adults with chronic diseases and needing coordinated care transitions from in-patient care to home. Data were analyzed using inductive qualitative content analysis.

**Results:**

The findings indicate that despite being the supposed main character, the older adult is not always involved in the planning and decision-making of their own care transition, often having poor insight and involvement in, and impact on, these aspects. This leads to an experience of mismatch between actual needs and the expectations of planned support after discharge.

**Conclusions:**

The study reveals a notable disparity between the assumed central role of older adults in care transitions and their insight and involvement in planning and decision-making.

**Supplementary Information:**

The online version contains supplementary material available at 10.1186/s12913-024-11039-3.

## Background

The proportion of the population aged 65 years and older has grown rapidly in recent decades in most countries worldwide [1–4]. At the same time, there has been an increase in those with chronic diseases. This poses a challenge for healthcare systems that are already under financial strain [5]. Older adults with chronic diseases often need continuous healthcare services from several different healthcare providers at once [1, 6], which is associated with higher healthcare utilization and costs [7]. The need for care is expected to increase further among older adults worldwide [8], and there are fewer individuals available to care for the growing number of older adults. This is considered one of the main challenges facing welfare societies today [9]. People with chronic diseases often need help from multiple care providers and different levels of care, as care is not always organized to meet their needs [6]. The transition from in-patient care to home creates risks for adverse events in older adults [10, 11], which may affect their experience of daily life after being discharged due to having unmet needs, medication worries, and experienced communication gaps [11]. Older adults are known to be vulnerable when being transferred between different settings and levels of care [12], especially when they have chronic [13] or multiple chronic diseases [14, 15], or when healthcare systems are fragmented [12, 16, 17], as this can lead to adverse events affecting the individual, such as prolonged periods of care or death [17]. Taken together, this has led to calls for healthcare services to ensure coordination and continuity of care in care transitions [6].

The provision of healthcare services is varied among and within different countries. The common aim is to promote equality in access to, and equity in the use of healthcare services [18, 19]. Hence, there is no universal model to use for integrated care, and local adaptations for this is used based on local needs [20, 21]. Therefore, at national level actions have been taken to facilitate care transitions and improve interactions between healthcare providers when delivering care. This has been done in Sweden by prioritizing coordination of care transitions from in-patient care to home [22] as well as an ongoing re-orientation of healthcare in Sweden and other OECD countries, in which primary care is supposed to be the basis for healthcare and the main point of contact for individuals [23]. The main goal is to better meet the needs of an aging population especially those with chronic conditions, as well as to enhance healthcare efficiency, diminish avoidable hospital admissions, and limit specialized care use. In spite of the aforementioned actions, healthcare systems remain fragmented [23, 24] and have poor coordination between different healthcare providers delivering care [25–27]. Healthcare providers have a duty to coordinate health- and social care services for older adults who need support upon discharge from in-patient care to out-patient care (primary care), municipal healthcare, and/or social services [22]. Besides this, older adults who need the support have a legal right to participate in planning for their health- and social care services [28–30], based on a democratic right to shape their formal care and support.

In previous studies focusing on older adults’ experiences of discharge from in-patient care, older adults have expressed a wish to be involved in their discharge [31–34], as well as in the decision-making regarding post-discharge care and support [32–36]. They have also raised concerns about not receiving support adapted to their individual needs upon discharge [32], or in case of future needs for support [32, 36]. Discharges from in-patient care to home have been studied for a long time [37–40], and previous research emphasizes the importance of continuity of care at discharge to prevent readmissions [41], as well as continuity of care interventions for preventing short-term hospital readmission among older adults with chronic diseases [42]. However, in the last decades, there has been an increased emphasis on patient involvement in clinical practice and in related healthcare legislations [28–30]. This involves sharing information, decision-making, and service delivery among the people needing care and support and their healthcare providers [19, 28–30] The ongoing movement advocates for person-centered care [43], which implies a shift from objectifying and seeing patients as passive recipients of care, to instead seeing them as active partners in care, where health systems respond to their individual needs and preferences [44].

Hence, there is a need to investigate whether these efforts have had an impact on clinical practice from the perspective of older adults. Therefore, the aim of the study was to describe older adults’ experiences of care transitions from in-patient care to home.

## Methods

### Study design and setting

The study had a qualitative approach with a descriptive design [45], drawing from general tenets of naturalistic inquiry. Chosen deliberately in seeking to describe older adults’ multiple experiences of care transitions from in-patient care to home. We obtained older adults experiences through individual interviews [46], and analyzed using inductive content analysis [47], allowing us to provide straight descriptions [45] and descriptive summaries of the older adults’ experiences, close to how they were described [48].

The study was performed in a region in the south of Sweden. The Swedish welfare system is mainly tax-funded, with a high degree of decentralization [49]. The responsibility for providing healthcare and social care services is distributed across 21 regions and 290 municipalities [44, 50]. The regions have responsibility for the funding and provision of in-patient care, specialist care, and primary care, whereas the municipalities have responsibility for providing home healthcare and social care in people’s homes [49]. National legislation regulates care transitions from in-patient care to home, and local adaptations are made by regions and municipalities based on their specific circumstances for coordinating discharge [22].

### Recruitment and data collection

Participants were purposefully sampled [51, 52]. We used a criterion sampling approach as we sought to provide information-rich descriptions [48] of older adults’ experiences of care transitions from in-patient care to home. We therefore had predetermined criterions for sampling in this study. According to the inclusion criteria, participants should be aged 65 years or older, communicate in Swedish, and have at least one chronic disease (e.g., heart failure, chronic obstructive pulmonary disease, stroke, or diabetes). They should also have a continued need for care and/or social support at home and their care transitions should be coordinated among multiple healthcare providers. Older adults diagnosed with general cognitive impairment or dementia were excluded from the study. Healthcare professionals who worked with coordination of care transitions in municipalities or at hospital wards assisted in identifying, verbally informing, and initially asking older adults about their interest in participating in the study. A total of 22 older adults initially agreed to participate. The first author then took the first contact with the older adults by phone. We had a multi-step process in which the older adults were provided with repeated information about the study, participation being voluntary, the possibility to ask questions, including the opportunity to withdraw participation anytime. This was crucial in our study because five of the older adults withdrew due to a change of mind (*n* = 2), as well as illness (*n* = 2), and being asked not to participate by close relatives (*n* = 1). Finally, 17 participants, five men and twelve women, aged between 65 and 92 years (mean = 81) were interviewed between January and June 2022. The participants were asked to choose a place and time for their interview. Twelve of the interviews were conducted face-to-face in the participants’ homes, and five were conducted by telephone, all on average 25 days after discharge. All participants gave their verbal and informed consent before the interviews began. The written consents in face-to-face interviews were signed before the interviews began, and in the telephone interviews, they were signed and sent to the first author after the interviews.

Each interview followed an interview guide (see Supplementary File 1), developed, and discussed collaboratively by the co-authors. The guide was designed to be as clear and unstructured as possible to obtain inductive data suitable for the aim of this study. The interviews started with an open-ended question encouraging the participants to talk freely about their experiences of being discharged. Other questions were geared towards collecting descriptions of how their discharge was planned and decided upon during their hospital stay, and about their experiences of returning home concerning their planned discharge. Probing questions were used, asking the participants to elaborate on their answers and give examples of their experiences. The length of the interviews varied between 22 and 70 min (mean = 37). All interviews were audio-recorded and transcribed verbatim, except one where the participant did not feel comfortable being audio-recorded. Instead, notes were taken and checked for consistency with the participant during, and after the interview. The analysis took place in parallel with the recruitment of older adults and data collection, during this process we observed saturation, indicating that no new information emerged [53].

### Data analysis

A qualitative content analysis with an inductive approach [47] was performed. Using a data-driven approach allowed us to explore patterns of described experiences within the interviews while remaining closely tied to their reported experiences, i.e., on the manifest level [47]. The process of analyzing data followed several steps, including preparation, organizing, and reporting of data, and whole interviews were used as units of analysis [47]. All interviews were transcribed and checked for their accuracy against the audio-recorded interviews excluding one. In this interview, notes were taken during the interview in a manner that aimed to capture the conversation in a format resembling transcribed audio recordings. This allowed us to broadly analyze the written interview in the same way as the transcribed interviews. We found no difference in the depth of the data based on data collection type, nor for the interview not being audio-recorded. All transcripts were read through several times to get an overall understanding of the content. The transcripts were then re-read while doing open coding. Five transcripts were independently coded, compared, and discussed by the first and last authors, to validate the coding process and promote consistency in further coding. The remaining transcripts were open-coded by the first author and the identified codes were then collected in a coding sheet in Microsoft Word. The first author did the initial grouping of codes into subcategories based on their similarities and differences. These subcategories were then discussed with the last author, revised, grouped, and abstracted into generic categories. Ultimately, a main category was identified. All authors were involved in discussions about the coding and abstraction process until an agreement was reached. Eleven subcategories were abstracted into four generic categories and one main category. The subcategories describe the content in the generic categories, and the main category provides an overall description of the meaning of the content.

## Results

**Fig. 1:**
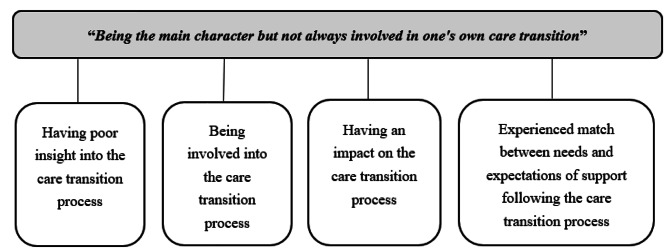
Overview of generic categories and main category

The analysis revealed one main category “*Being the main character but not always involved in one’s own care transition*”, and four generic categories. The first three categories describing the experiences of care transitions while hospitalized, and the fourth describing the older adults’ experiences after being discharged (Fig. 1). The experiences of the older adults varied, indicating diverse levels of involvement and influence in their care transition processes, as well as to what degree they experienced their needs and expectations being met after being discharged. Below, the results are presented with the sub-categories interwoven into the overall presentation of the generic categories.

### Having poor insight into the care transition process

The older adults described diverse experiences of how they had poor insight into their care transition process. They described themselves being provided with information about the care transition – in verbal and/or written form. However, this information was sometimes inadequate or not provided at all. Information regarding support and discharge time was usually given to the older adults in verbal form. Medical information such as changes in prescribed medication were often located in their list of medication, often not verbally informed by the healthcare professionals. Discharge notices, and exercise prescriptions were primarily given in written form. The written information was not always adapted to individual needs, and therefore not always understood or possible to assimilate when being unable to read due to impaired eyesight. Sometimes, the older adults described their experience of having diminished health or own acceptance of needing support as perceived obstacles for being involved in planning and decision-making. Poor insight also included being notified about the time of discharge, often the day before or the same day as discharge. This was experienced as too short notice and led to feelings of uncertainty, perceiving the discharge as being rushed, and not being ready or prepared to go home, as well as losing a sense of control over their situation.

*“’You can go home today. There’s a car in ten minutes,’ they said to me. ‘I can’t,’ I said, and that created a kind of awkward mood. But I think I remember that they came from home help services when I came home, but there I was without any food when I came home, and a little dazed.”* (Woman, 93).

Poor insight was also shown where the older adults described leaving the hospital only being informed about, and only knowing that someone from the home help services would meet them upon arrival. Some older adults thought that support provided at home was given routinely. There were also those older adults who described having poor insight in their care transition process by being unaware that a coordination process had taken place:

*“I don’t know anything about that [discharge planning].”* (Woman, 92).

This could be due to not understanding that they had attended their care plan meeting or not knowing either how or by whom their support had been planned and decided. Poor insight into the care transition process could also mean not being asked for their views on the perceived situation and need for support when coming home:

*“I was the main character in all this, really […] but they were the ones that planned most of it and then you just tagged along.”* (Woman, 76).

It could also involve not getting the choice to participate or exert personal influence over planned support – sometimes the planned support was experienced as predetermined by healthcare professionals and next of kin.

*“Yeah, when I say options, I think it’s pretty lousy to just force something on a person, you know, without asking: ‘where do you live and how to do you live?’ Nobody asked me that question, they didn’t care about that, I was supposed to walk along their path and that was a matter of course.”* (Male, 88).

Having poor insight into the care transition could also relate to leaving the hospital without being aware of planned support or knowing only that support was going to be provided, but not how or when.

*“No, I didn’t really know that [what support] at the time, no, no, I actually didn’t know that. But I knew that I would get all the help I needed, because it was the aid worker who called me at the hospital and she said that: ‘You can get all the help you need, X,’ she said, and I was very grateful for that, that they came several times a day if I needed it, and I knew that I had the help that …so I knew that I could get that, but it wasn’t really planned at the time.”* (Woman, 92).

There were also older adults who felt that the lack of information about when support was planned to be given at home contributed to poor insight into their care transition, leading to the experiences of the care transition process as being unplanned and uncertain. Although not being involved in the planning process between healthcare professionals and their next of kin, some older adults experienced having some insight into their care transition process. This was also experienced when having previous experiences of coordinated care transitions, or when being informed about the planned period of care and therefore knowing what to expect.

### Being involved into the care transition process

Being involved in the care transition process was mainly described as being invited by healthcare professionals to share one’s personal views on the care transition. The older adults described their views and need for support at home being inquired into during their hospitalization in various ways. For some of the older adults, this was experienced when they were invited to and had face-to-face meetings, or spontaneous talks on the hospital ward with various healthcare professionals – such as occupational therapists, physiotherapists, dieticians, and/or aid workers – about the perceived need for support at home.

*“Yeah, they ask about that, if you have the resources to manage on your own and if anyone checks on you and they ask if you have home help services or a next of kin that comes over and so on. So they are very, very attentive to that, they really are.”* (Male, 76).

The older adults felt involved when being invited to join conversations regarding their personal perception of their situation and need for support at home. Involvement could also mean participating and engaging in conversations that led to the decision of having a coordinated care plan meeting at the hospital before discharge. One older adult described her experience of being invited into such a conversation:

*“Yes, I got to answer questions, where I said what I thought.”* (Woman, 87).

Others described feeling involved when they were prepared and informed before discharge. For example, this could mean being educated in self-care before discharge, by learning how to administer medication on their own.

*“Yeah, there was a bit of reasoning about that, we tested out having me take my medication on my own and that kind of thing, so I could get used to it. Of course, I could do that before I went to hospital, so it wasn’t that big a difference. I’m diabetic, so I take insulin.”* (Male, 66).

The older adults also felt involved when they were invited to talk about their expected level of independence and the support, they felt would be reasonable after discharge, or when having preparatory and coordinated home visits to review the situation at home together with healthcare professionals from the municipality and in-patient care before discharge. It was also experienced when being invited to attend the coordinated care plan meeting or being informed that a coordinated care planning meeting would take place at home after discharge.

### Having an impact on the care transition process

The older adults mainly described having an impact on their care transition process as having a choice of being involved in the planning and deciding upon the support to be provided after discharge. There were also older adults who felt they had an impact when they were listened to and had their requests for support approved by healthcare professionals:

*“Yeah, you could say that I have those requests [for support], of course, and I’ve had them approved, if I can use that word.”* (Woman, 92).

Some older adults felt that they had an impact on the care transition process through self-determination and having a direct influence over planned support, including being able to refuse support if asked about their perceived needs for support after discharge.

*“No, I don’t know if we talked about that. I guess they asked if I needed any more support or more help, but I said that I don’t need that, what I have is enough.”* (Woman, 79).

There were also older adults who described that they felt having an impact in their care transition as they actively handed over responsibility to others to plan and decide for support in their place. This was due to various reasons, such as not seeing their own participation as important, perceiving their health and energy during hospitalization as lacking, or trusting and feeling safe that healthcare professionals and next of kin planned for their support. For others, the longing to come home was greater than the desire to participate in the planning and decision-making in the care transition. Having faith that support would work at home as it had before the hospital admission was also described as a reason to hand over responsibility. Having an impact could also mean accepting the support that healthcare staff and next of kin had planned only until it no longer felt necessary:

*“Because I said ‘I’ll accept it as long as I feel that I need it,’ I said, and it was nice, as they had said.”* (Woman, 75).

### Experienced match between needs and expectations of support following the care transition process

The older adults described various experiences of how their planned support had matched their perceived needs and expectations at home, regardless of their experiences of having insight, being involved, or having an impact on the care transition. Those who were pleased with the support they had been provided at home described receiving support corresponding to their experienced needs or receiving more support than they thought they needed:

*“Yes, oh yes, oh yes, more [support] than what was really needed, but that’s how it is. It was really nice.”* (Woman, 75).

Not being invited to be involved in planning or decision-making regarding future support at home or choosing not to be involved also led to experiences of not receiving support matching their experienced individual needs. This could mean getting less support than needed or being discharged with support that was not needed. Some described having an altered need for support, for instance, that their need for support had decreased due to being more independent in managing everyday life after having spent some time at home. The support provided at home was sometimes described as malfunctioning in various ways. A complete or partial mismatch between needs and expectations of support upon returning home could appear due to coordination issues among healthcare professionals and/or insufficient resources in the municipalities. There were also older adults who described not understanding or being aware that support would not be provided immediately upon their return home, or that further planning would take place only when they had spent several days at home.

*“The first week when I was back home from hospital, it didn’t work and I didn’t know anything, like how or when they’d show up. […] Coming home felt unsafe, I had never experienced a week like that. I didn’t really know what to expect when I got home. My son drove me home and, well, there I was.”* (Woman, 93).

For some older adults, the planned support did not meet their expectations of what planned support should entail. Therefore, the support did not match their expectations or correspond to their perceived needs for support:

*“No, I had expected a bit more to happen there […], I wish that the home help services had shown up sooner.”* (Woman, 74).

Having a follow-up of the post-discharge support was considered crucial by those for whom support had not worked out or was less than expected. After being discharged, some older adults had follow-ups – usually at the hospital, in primary care, or with nurses from municipality healthcare who had given them targeted support, such as wound care or medication reviews. Most of the older adults described not having had any structured follow-up of the post-discharge support, especially not of their social support at home. A follow-up of social support was usually only performed when such support had a time limit.

## Discussion

This study aimed to describe older adults’ experiences of care transitions from in-patient care to home. It revealed that despite being the supposed main characters in care transitions, older adults often experienced poor insight, involvement, and impact in their care transition process, which is in line with the results of other studies [39]. One of the reasons for not being involved was experiencing impaired health, which studies by others confirm [54–56]. However, we also saw that the older adults’ lack of involvement in care transitions was not always their own choice, but also depended on the degree to which they were invited to be involved, which is supported by previous research [57]. The involvement of patients has been argued to be co-determined among patients and healthcare professionals, occurring only when there are mutual relationships of dialogue and shared decision-making [58]. Ebrahimi et al. [59] found that co-creation with the patient is fundamental for the implementation of person-centered care in an out-of-hospital setting. Despite evidence that adopting a person-centered approach enhances the discharge process and reinforces the perception of patients as capable of actively participating in their care planning [60], both our and previous studies indicate that older adults are not always invited to share their perspectives [33, 61]. Our study highlights the importance of recognizing and addressing the diverse needs of older adults to be involved and have impact in their care transition. Also Nilsen et al. [62] found that older adults prefer to be involved in various degrees in decision-making when being discharged from in-patient care to home. Our results also suggest that the care transition can, from the part of the older adults, be understood as a lack of collaborative shared decision-making, only involving healthcare professionals and not the older adults. This is in line with previous findings from the perspective of healthcare professionals and citizens [58] and aligns with similar insights from a study focusing on quality enhancements in healthcare, in which the focus in practice was to, for, or with patients and families [63].

The limited involvement of older adults in their care transition process when being discharged from in-patient care to home can also be due to health professionals focusing on care coordination over patients’ involvement [64–69]. Adhering to set frameworks for the discharge process and considering discharge planning meetings as a place for professional exchange rather than a place for dialogue where older adults are also included [67]. Poor insight into the care transitions is in direct contradiction with current legislation, where the individual’s democratic right to exercise influence over their healthcare and social care is emphasized and should guide the design of the provided support [28–30]. In our study, some older adults were not asked to share their personal views on the care transition, nor their need for support after discharge, although being asked to share personal views was considered fundamental to feeling involved in the care transition. Similar findings indicate that older adults are frequently not encouraged to discuss their main goals with healthcare professionals when managing chronic conditions. Additionally, they also found gaps identified in hospital discharge planning, particularly concerning the absence of written discharge plans [70].

The older adults also described experiences of having insufficient information, sometimes not adapted to their individual needs. If information is available, a patient who is discharged from inpatient care must receive information about existing plans for ongoing care and for their care after discharge [22]. The person providing the information must ensure that the recipient has understood the content and meaning, and if necessary, provide the information in writing. The insufficient information mainly concerned the discharge process, planned support, and medications. Previous research has shown that patients get insufficient information about medication, both in care [39, 61] and at discharge [18, 71]. The older adults mentioned how most of the provided information was given verbally. In our study, we do not know what information regarding the care transition was de facto provided, but a perceived lack of provided information and poor insight into their care transition process left the older adults with a feeling of uncertainty upon returning home. This is consistent with previous research showing that if information is provided without confirming that it is understood, patients are left feeling uncertain [18]. When information is provided both verbally and in writing at discharge, older adults feel well-informed [34]. This underlines the need to provide both verbal and written information – verbal information is often forgotten [34, 54, 72–74]. In addition, information and knowledge exchange between healthcare professionals and an older adult is shown to strengthen the feeling of being involved in one’s own discharge [34]. In other care contexts, being listened to has been shown to be important for patients to experience participation [65] and it has previously been shown that older adults want to be involved in their own care [31–34, 39, 66] and participate in decision-making at discharge [32, 33, 35, 36, 75].

In our study, the older adults described different experiences of how planned support was consistent with and adapted to their perceived needs and expectations of support in the home. Similar findings have been made regarding older adults with chronic illnesses [12], who have expressed concerns about not getting help tailored to their individual needs [33, 37]. This may be because help is not always tailored to the individual’s perceived needs [11] or because nurses need to balance the patient’s legal right to self-determination against what they consider to be appropriate care at discharge [76]. Research suggests that there is a need to focus on patients’ needs while also standardizing the tools used, to ensure both quality and coordination in integrated care [54]. Other studies have pointed out the importance of targeting patients’ needs at discharge to prevent readmissions [77, 78], also, the positive effects of discharge planning in readmissions [79, 80], especially when combined with follow-ups [81]. Our study indicates that there is a notable difference between older adults assumed central role in care transitions and their level of insight and involvement in planning and decision-making. The findings showed that the involvement and influence of older adults in their care transition process still very much is a process of communication among healthcare professional, and not primarily a collaborative shared decision-making process involving the older adults themselves. Hence, the position of older adults in care transitions from in-patient care to home does not yet seem to have changed in the Swedish context, despite previous efforts to strengthen the patient’s position [66, 82].

### Strengths and limitations

This study has both strengths and limitations that should be considered when interpreting the results. Strength lies in the practical experience of the first author, who has worked with care transitions for older adults as a social worker for many years, providing valuable insights into the study’s context. Complemented by the interdisciplinary composition of the research team and their diverse backgrounds in social work and healthcare, each member’s unique perspective enriched the research process, interview-guide development, and facilitated for a nuanced data analysis. A comprehensive description of context, participant characteristics, research methodology, and findings were provided together with several quotations from the older adults to provide transparency and to enhance trustworthiness in this study. A qualitative descriptive design was deliberately chosen as we were interested in experiences of care transitions from the perspective of older adults. We considered interviews to be the most appropriate data collection method, combined with purposefully sampling our participants using a criterion approach. Limitations arise primarily from sampling constraints, as we excluded older adults with cognitive impairments or diagnosed with dementia, reducing the risk of recall bias and for ethical reason, as we asked questions about their experiences of an event that had already passed in time. An inclusion criterion was that the older adults were Swedish speaking, this might have excluded older adults with diverse cultural and linguistic backgrounds and overlooked their perspective.

## Conclusion

In conclusion, our study reveals a notable disparity between the assumed central role of older adults in the care transition process and their described experiences of levels of involvement and influence in planning and decision-making. Our findings underscore the pivotal role of healthcare professionals in actively involving older adults throughout the care transition process when being discharged from in-patient care to home. This includes incorporating the patient’s preferences for involvement in and influence on planning and decision-making, to ensure that planned support aligns with their individual needs and preferences. By involving the older adult, acknowledging their preferences and ensuring timely and clear information, the care transition process from in-patient care to home, will be more individualized facilitating seamless care transitions where the older adults are the main character in their own care transition.

### Electronic supplementary material

Below is the link to the electronic supplementary material.

Supplementary Material 1

## Data Availability

No datasets were generated or analysed during the current study.

## References

[1] World Health Organization. World report on ageing and health. 2015. [Internet]. [cited 2023 Dec 19]. https://www.who.int/publications/i/item/9789241565042.

[2] Organisation for Economic Co-operation and Development. Demographic references: population age structure 2022. [Internet]. [cited 2023 Dec 19]. https://stats.oecd.org/Index.aspx?QueryId=30142.

[3] United Nations, Ageing. 2023. [Internet]. [cited 2023 Dec 19]. https://www.un.org/en/global-issues/ageing.

[4] Organisation for Economic Co-operation and Development. Demographic references: population age structure 2023. [Internet]. [cited 2023 Dec 19]. https://stats.oecd.org/index.aspx?queryid=30130.

[5] Muka T, Imo D, Jaspers L, Colpani V, Chaker L, van der Lee SJ (2015). The global impact of non-communicable diseases on healthcare spending and national income: a systematic review. Eur J Epidemiol.

[6] Coleman EA, Boult C (2003). Improving the quality of transitional care for persons with complex care needs. J Am Geriatr Soc (JAGS).

[7] Marengoni A, Angleman S, Melis R, Mangialasche F, Karp A, Garmen A (2011). Aging with multimorbidity: a systematic review of the literature. Ageing Res Rev.

[8] Organisation for Economic Co-operation and Development. New job opportunities in an ageing society 2019. [Internet]. [cited 2023 Dec 19]. http://www.oecd.org/g20/summits/osaka/ILO-OECD-G20-Paper-1-3-New-job-opportunities-in-an-ageing-society.pdf.

[9] National Board of Health and Welfare. Vård och omsorg om äldre - Lägesrapport 2022. [Internet]. [cited 2023 Dec 19]. https://www.socialstyrelsen.se/globalassets/sharepoint-dokument/artikelkatalog/ovrigt/2022-3-7791.pdf; 2022 Report No.: 2022-3-7791.

[10] Laugland KA. Transitional care of the elderly from a resilience perspective. [licentiate thesis on the internet]. Norway: University of Stavanger; 2015.

[11] Andreasen J, Lund H, Sørensen EE. The experience of daily life of acutely admitted frail elderly patients one week after discharge from the hospital. Int J Qualitative Stud Health Well-being. 2015;10(1). 10.3402/qhw.v10.27370 PMC4452652 26037333

[12] Cline DD (2016). A concept analysis of vulnerability during transitions. Nurs Educ Perspect.

[13] Allen J, Hutchinson AM, Brown R, Livingston PM (2017). User experience and care integration in transitional care for older people from hospital to home: a meta-synthesis. Qual Health Res.

[14] Craven E, Conroy S (2015). Hospital readmissions in frail older people. Reviews Clin Gerontol.

[15] Summer Meranius M. Era delar är min helhet: En studie om att vara äldre och multisjuk. [licentiate thesis on the internet]. Växjö: Linnaeus University; 2010.

[16] Leppin AL, Gionfriddo AR, Kessler M, Brito JP, Mair FS, Gallacher K (2014). Preventing 30-day hospital readmissions: a systematic review and meta-analysis of randomized trials. JAMA Intern Med.

[17] Schildmeijer KGI, Unbeck M, Ekstedt M, Lindblad M, Nilsson L (2018). Adverse events in patients in home healthcare: a retrospective record review using trigger tool methodology. BMJ Open.

[18] Trydegård GB, Thorslund M (2010). One uniform welfare state or a multitude of welfare municipalities? The evolution of local variation in Swedish elder care. Social Policy Adm.

[19] World Health Organization. Integrated people-centred care 2023. [Internet]. [cited 2023 Dec 19]. https://www.who.int/health-topics/integrated-people-centered-care#tab=tab_2.

[20] Goodwin N, Sonola L, Thiel V, Kodner D. Co-ordinated care for people with complex chronic conditions - key lessons and markers for success. [Internet]. 2013. [cited 2023 Dec 19]. https://www.kingsfund.org.uk/publications/co-ordinated-care-people-complex-chronic-conditions.

[21] Wodchis WP, Dixon A, Anderson GM, Goodwin N (2015). Integrating care for older people with complex needs: key insights and lessons from a seven-country cross-case analysis. Int J Integr Care.

[22] Ministry of Social Affairs. Lag. (2017:612) om samverkan vid utskrivning från sluten hälso- och sjukvård. [Internet]. [cited 2023 Dec 19]. https://www.riksdagen.se/sv/dokument-och-lagar/dokument/svensk-forfattningssamling/lag-2017612-om-samverkan-vid-utskrivning-fran_sfs-2017-612/.

[23] OECD. Realising the potential of primary health care. 2020. [Internet]. [cited 2023 Dec19]. https://www.oecd.org/health/realising-the-potential-of-primary-health-care-a92adee4-en.htm.

[24] Hansson A, Svensson A, Ahlström BH, Larsson LG, Forsman B, Alsén P (2018). Flawed communications: health professionals’ experience of collaboration in the care of frail elderly patients. Scand J Public Health.

[25] OECD. Who cares? Attracting and retaining care workers for the elderly 2020. [Internet]. [cited 2023 Dec 19]. https://www.oecd.org/publications/who-cares-attracting-and-retaining-elderly-care-workers-92c0ef68-en.htm.

[26] National Board of Health and Welfare. Uppföljning av omställningen till en mer nära vård 2020 - Utvecklingen i regioner och kommuner samt förslag på indikatorer 2020. [Internet]. [cited 2023 Dec 19]. https://www.socialstyrelsen.se/globalassets/sharepoint-dokument/artikelkatalog/ovrigt/2021-8-7496.pdf.

[27] Swedish Agency for Health and Care Services Analysis. Nationell uppföljning av hälso- och sjukvården 2022. Indikatorer på kvalitet, jämlikhet och effektivitet. 2022. [Internet]. [cited 2023 Dec 19]. https://www.vardanalys.se/rapporter/nationell-uppfoljning-av-halso-och-sjukvarden-2022/ Report No.: PM 2022:3.

[28] Ministry of Social Affairs. Patientlag. (2014:821). [Internet]. [cited 2023 Dec 19]. https://www.riksdagen.se/sv/dokument-och-lagar/dokument/svensk-forfattningssamling/patientlag-2014821_sfs-2014-821/.

[29] Ministry of Social Affairs. Socialtjänstlag. (2001:453). [Internet]. [cited 2023 Dec 19]. https://www.riksdagen.se/sv/dokument-och-lagar/dokument/svensk-forfattningssamling/socialtjanstlag-2001453_sfs-2001-453/.

[30] Ministry of Social Affairs. Hälso- och sjukvårdslag (2017:30). 2017. [Internet]. [cited 2023 Dec 19]. https://www.riksdagen.se/sv/dokument-och-lagar/dokument/svensk-forfattningssamling/halso-och-sjukvardslag-201730_sfs-2017-30/.

[31] Foss C, Hofoss D (2010). Elderly persons’ experiences of participation in hospital discharge process. Patient Educ Couns.

[32] Gabrielsson-Järhult F, Nilsen P (2016). On the threshold: older people’s concerns about needs after discharge from hospital. Scand J Caring Sci.

[33] Jobe I, Lindberg B, Nordmark S, Engström Å (2018). The care-planning conference: exploring aspects of person‐centred interactions. Nurs Open.

[34] Schjødt K, Erlang AS, Starup-Linde J, Jensen AL (2022). Older hospitalised patients’ experience of involvement in discharge planning. Scand J Caring Sci.

[35] Storm M, Siemsen IMD, Laugaland K, Dyrstad DN, Aase K (2014). Quality in transitional care of the elderly: key challenges and relevant improvement measures. Int J Integr Care.

[36] Harrefors C, Sävenstedt S, Axelsson K (2009). Elderly people’s perceptions of how they want to be cared for: an interview study with healthy elderly couples in Northern Sweden. Scand J Caring Sci.

[37] Groene RO, Orrego C, Suñol R, Barach P, Groene O (2012). It’s like two worlds apart: an analysis of vulnerable patient handover practices at discharge from hospital. BMJ Qual Saf.

[38] Naylor M, Brooten D, Jones R, Lavizzo-Mourey R, Mezey M, Pauly M (1994). Comprehensive discharge planning for the hospitalized elderly: a randomized clinical trial. Ann Intern Med.

[39] Dyrstad D, Testad I, Aase K, Storm M (2015). A review of the literature on patient participation in transitions of the elderly. Cogn Technol Work.

[40] Flink M, Ekstedt M (2017). Planning for the discharge, not for patient self-management at home – an observational and interview study of hospital discharge. Int J Integr Care.

[41] Säfström E, Årestedt K, Liljeroos M, Nordgren L, Jaarsma T, Strömberg A (2023). Associations between continuity of care, perceived control and self-care and their impact on health-related quality of life and hospital readmission-a structural equation model. J Adv Nurs.

[42] Facchinetti G, D’Angelo D, Piredda M, Petitti T, Matarese M, Oliveti A (2020). Continuity of care interventions for preventing hospital readmission of older people with chronic diseases: a meta-analysis. Int J Nurs Stud.

[43] Ekman I, Swedberg K, Taft C, Lindseth A, Norberg A, Brink E, Carlsson J, Dahlin-Ivanoff S, Johansson I-L, Kjellgren K, Lidén E, Öhlén J, Olsson L-E, Rosén H, Rydmark M, Sunnerhagen KS (2011). Person-centered Care — Ready for Prime Time. Eur J Cardiovasc Nursing: J Working Group Cardiovasc Nurs Eur Soc Cardiol.

[44] Ministry of Social Affairs. God och nära vård – En primärvårdsreform. (SOU 2018:39) [Internet]. 2018 Jun 01 [cited 2023 Dec 19]. https://www.regeringen.se/rattsliga-dokument/statens-offentliga-utredningar/2018/06/sou-201839/.

[45] Sandelowski M (2000). Whatever happened to qualitative description?. Res Nurs Health.

[46] Brinkmann S, Kvale S. Doing interviews. 2nd edition. London: SAGE Publications Ltd; 2018.

[47] Elo S, Kyngäs H (2008). The qualitative content analysis process. J Adv Nurs.

[48] Polit D, Beck CH. Nursing research: generating and assessing evidence for nursing practice. Eleventh edition. Wolters Kluwer; 2021.

[49] Anell A, Glenngård AH, Merkur S (2012). Sweden: health system review. Health Syst Transition.

[50] Bergmark Å, Minas R. Decentraliserad välfärd eller medborgerliga rättigheter? Om omfördelning av makt och ansvar mellan stat och kommun. *Socialvetenskaplig tidskrift*. 2016;14(2–3 (2007).

[51] Patton MQ (2015). Qualitative research & evaluation methods: integrating theory and practice.

[52] Sandelowski M (2010). What’s in a name? Qualitative description revisited. Res Nurs Health.

[53] Malterud K, Siersma VD, Guassora AD. Sample size in qualitative interview studies: guided by Information Power. Qual Health Res.2015;pii:1049732315617444[Epub ahead of print]. 10.1177/1049732315617444 26613970

[54] Lilleheie I, Debesay J, Bye A, Bergland A (2019). Experiences of elderly patients regarding participation in their hospital discharge: a qualitative metasummary. BMJ Open.

[55] Lindberg C, Fagerström C, Willman A (2018). Patient autonomy in a high-tech care context—a theoretical framework. J Clin Nurs.

[56] Hardicre N, Murray J, Shannon R (2021). Doing involvement: a qualitative study exploring the ‘work’ of involvement enacted by older people and their carers during transition from hospital to home. Health Expect.

[57] Lemetti T, Voutilainen P, Stolt M, Eloranta S, Suhonen R (2019). Older patients’ experiences of nurse-to‐nurse collaboration between hospital and primary health care in the care chain for older people. Scand J Caring Sci.

[58] Thompson AGH (2007). The meaning of patient involvement and participation in health care consultations: a taxonomy. Soc Sci Med.

[59] Ebrahimi Z, Patel H, Wijk H, Ekman I, Olaya-Contreras P (2021). A systematic review on implementation of person-centered care interventions for older people in out-of-hospital settings. Geriatric Nurs (New York).

[60] Ulin K, Olsson L-E, Wolf A, Ekman I (2016). Person-centred care – an approach that improves the discharge process. Eur J Cardiovasc Nursing: J Working Group Cardiovasc Nurs Eur Soc Cardiol.

[61] Erlang AS, Schjødt K, Linde JKS, Jensen AL (2021). An observational study of older patients’ experiences of involvement in discharge planning. Geriatr Nurs.

[62] Nilsen ER, Hollister B, Söderhamn U, Dale B (2022). What matters to older adults? Exploring person-centred care during and after transitions between hospital and home. J Clin Nurs.

[63] Suutari A-M, Thor J, Nordin AMM, Kjellström S, Areskoug Josefsson K (2021). Improving health for people living with heart failure: focus group study of preconditions for co-production of health and care. J Participatory Med.

[64] Swedish Agency for Health and Care Services Analysis. Laga efter läge - Uppföljning av lagen om samverkan vid utskrivning från slutenvården. 2020. [Internet]. [cited 2023 Dec 19]. https://www.vardanalys.se/rapporter/laga-efter-lage/ Report No.: 2020:4.

[65] Eldh AC, Ekman I, Ehnfors M (2010). A comparison of the concept of patient participation and patients’ descriptions as related to healthcare definitions. Int J Nurs Knowl.

[66] Swedish Agency for Health and Care Services Analysis. En lag som kräver omtag. 2021. [Internet]. [cited 2023 Dec 19]. https://www.vardanalys.se/rapporter/en-lag-som-kraver-omtag/ Report No.: 2021:10.

[67] Bångsbo A, Duner A, Dahlin-Ivanoff S, Liden E (2017). Collaboration in discharge planning in relation to an implicit framework. Appl Nurs Res.

[68] Mabire C, Büla C, Morin D, Goulet C (2015). Nursing discharge planning for older medical inpatients in Switzerland: a cross-sectional study. Geriatr Nurs.

[69] Kneck Å, Flink M, Frykholm O, Ekstedt M (2019). The information flow in a healthcare organisation with integrated units. Int J Integr Care.

[70] Osborn R, Moulds D, Squires D, Doty MM, Anderson C (2014). International survey of older adults finds shortcomings in access, coordination, and patient-centered care. Health Aff.

[71] Norberg S, Gustafsson M (2018). Older peoples’ adherence and awareness of changes in drug therapy after discharge from hospital. Pharmacy.

[72] Horstman MJ, Mills WL, Herman LI, Cai C, Shelton G, Qdaisat T (2017). Patient experience with discharge instructions in postdischarge recovery: a qualitative study. BMJ Open.

[73] Kang E, Gillespie BM, Tobiano G, Chaboyer W (2018). Discharge education delivered to general surgical patients in their management of recovery post-discharge: a systematic mixed studies review. Int J Nurs Stud.

[74] Saunders DD, Dineen D, Gullick K, Seaman K, Graham R, Finlay S (2022). Exploring orthopaedic patients’ experiences of hospital discharge: implications for nursing care. Collegian.

[75] Dyrstad DN, Laugaland KA, Storm M (2015). An observational study of older patients’ participation in hospital admission and discharge - exploring patient and next of kin perspectives. J Clin Nurs.

[76] Gustafsson L-K, Zander V, Bondesson A, Pettersson T, Anbacken E-M, Östlund G (2022). Actions taken to safeguard the intended health care chain of older people with multiple diagnoses - a critical incident study. BMC Nurs.

[77] González-Ortiz L, Calciolari S, Goodwin N, Stein V. The Core Dimensions of Integrated Care: A literature review to support the development of a Comprehensive Framework for Implementing Integrated Care. Int J Integr Care. 2018;18(3). 10.5334/ijic.4198 PMC6137610 30220893

[78] Flink M. Patients’ position in care transitions: an analysis of patient participation and patient-centeredness. Karolinska institutet; 2014.

[79] Gonçalves-Bradley DC, Lannin NA, Clemson LM, Cameron ID, Shepperd S (2016). Discharge planning from hospital. Cochrane Database Syst Reviews.

[80] Hunt-O’Connor C, Moore Z, Patton D, Nugent L, Avsar P, O’Connor T (2021). The effect of discharge planning on length of stay and readmission rates of older adults in acute hospitals: a systematic review and meta‐analysis of systematic reviews. J Nurs Adm Manag.

[81] Henke RM, Karaca Z, Jackson P, Marder WD, Wong HS. M: MCRR, 345–368. Discharge Planning and Hospital Readmissions. *Medical care research and review*. 2017;74(3):345 – 68. 10.1177/1077558716647652 27147642

[82] Swedish Agency for Health and Care Services Analysis. Act without impact. 2017. [Internet]. [cited 2023 Dec 19]. https://www.vardanalys.se/in-english/reports/act-without-impact/ Report No.: 2017:2.

[83] World Medical Association. WMA DECLARATION OF HELSINKI – ETHICAL PRINCIPLES FOR MEDICAL RESEARCH INVOLVING HUMAN SUBJECTS. 2022 Sep. [Internet]. [cited 2023 Dec 19]. https://www.wma.net/policies-post/wma-declaration-of-helsinki-ethical-principles-for-medical-research-involving-human-subjects/.

[84] Regulation (EU). 2016/679 of the European Parliament and of the Council of 27 April 2016 on the protection of natural persons with regard to the processing of personal data and on the free movement of such data, and repealing Directive 95/46/EC (General Data Protection Regulation) [Internet]. [cited 2023 Dec 19]. http://data.europa.eu/eli/reg/2016/679/oj.

