# Supplementary material for: “Being the main character but not always involved in one’s own care transition” - a qualitative descriptive study of older adults’ experiences of being discharged from in-patient care to home
Source: BMC Health Serv Res. 2024 May 2;24:571. doi: 10.1186/s12913-024-11039-3 (PMC11067295; doi:10.1186/s12913-024-11039-3)
Supplement: Supplementary file 1 — Supplementary Material 1 [file 12913_2024_11039_MOESM1_ESM.docx]

### SUPPLEMENTARY FILE: Interview guide

1. Could you tell me about your experiences of being discharged from the hospital to your home?

- How was your discharge planned?
- What information did you receive about the planning for your discharge?
- Were you involved in your discharge planning? e.g., physical meetings, video meetings, phone calls?
- In what ways were you involved in determining the help you would receive when you got home?

1. Could you tell me about your experiences of what happened when you arrived home from the hospital?

- Please share both positive/negative experiences.
- Did the help you received match what was planned?

1. Did you receive any paperwork about your discharge?

If yes:

- What would you say the papers you received from the hospital were about?
- Was there any information that you felt was missing, if so, what?

If no:

- Have you thought of any written information that you would have liked to take home with you?

1. Has there been any follow-up on the support/assistance you received at home since you came back from the hospital?
2. Was there anything in your discharge process that could have been done differently, during your hospital stay or after you returned home? If so, what?
3. Based on what we have talked about during the interview, is there anything of importance to you that we haven't discussed?
